# Understanding acceptance of digital smoking cessation interventions: user behavior, key influencing factors, and the role of reimbursement

**DOI:** 10.1186/s12889-025-25472-4

**Published:** 2025-12-12

**Authors:** Franziska Theile, Tom Schaal, Melanie Mäder, Tonio Schönfelder

**Affiliations:** 1 Faculty of Health and Healthcare Sciences, University of Applied Sciences Zwickau, Kornmarkt 1, Zwickau, 08066 Germany; 2 Scientific Institute for Health Economics and Health Services Research (WIG2 GmbH), Puschstr. 6a, Leipzig, 04103 Germany; 3 Faculty of Economics and Management Science, Chair for Health Economics and Management, Leipzig University, Grimmaische Str. 12, Leipzig, 04109 Germany; 4 Chair of Health Sciences – Public Health, Dresden University of Technology, Fetscherstr. 74, 01307 Dresden, Germany

**Keywords:** DiGA, digital smoking cessation intervention, Self-Efficacy, Technology acceptance, UTAUT2, Willingness to pay

## Abstract

Smoking remains a prominent preventable health risk in Germany, creating a need for effective cessation interventions. Digital smoking cessation interventions (DSCIs) present promising support for individuals aiming to quit, yet their utilization and acceptance are not thoroughly understood. This study analyzes usage patterns and acceptance levels of DSCIs among smokers, occasional smokers, and former smokers in Germany, focusing on user behavior, acceptance determinants, and the influence of prescription and reimbursement status. An online questionnaire based on the Unified Theory of Acceptance and Use of Technology 2 (UTAUT2) model was administered to participants. Data were collected through recruitment via social media, email lists, counseling groups, and public postings. The responses were analyzed using SPSS. The study included 173 participants (61.85% female, 37.57% male, 0.58% diverse) with an average age of 35.28 years. They reported smoking for an average of 18.21 years and attempting cessation 3.42 times. Among respondents, 41.62% had used DSCIs, predominantly former smokers (54.17%) and women (79.17%), with the “Smoke Free” app being the most utilized intervention. Although 73.05% expressed willingness to (re)use DSCIs, actual usage showed moderate acceptance levels. Significant predictors of acceptance included willingness to pay (*p* = 0.013), self-efficacy (*p* = 0.018), and physician prescription with clinical evidence (*p* = 0.019). The results highlight a rising demand for digital solutions focused on long-term smoking cessation, particularly among middle-aged women, emphasizing the need for a deeper understanding of acceptance drivers and model expansions to address healthcare dynamics.

**Supplementary Information:**

The online version contains supplementary material available at 10.1186/s12889-025-25472-4.

## Introduction

Smoking represents one of the most significant preventable risk factors for severe illnesses and premature mortality in industrialized nations. Tobacco kills more than 8 million people worldwide each year, including an estimated 1.3 million non-smokers exposed to second-hand smoke [1]. In 2018, smoking accounted for an estimated 127,000 deaths in Germany and was associated with approximately €30 billion in direct healthcare expenditures and an additional €67 billion in productivity losses [2, 3].

Despite proven health benefits and the availability of cessation programs funded by German statutory health insurance, participation remains low. In 2019, only 9,360 insured individuals used such programs—representing just 0.05% of the smoking population [4].

Given these challenges, digital health solutions are gaining importance. Digital interventions offer accessible, flexible, and cost-effective alternatives, integrating features such as savings tracking, motivational support, craving management, and health improvement insights [5], and can be categorized into freely available apps and prescription-based Digital Health Applications (“Digitale Gesundheitsanwendungen”, DiGA). Freely available apps can be downloaded without prescriptions and offer various supportive functions [6]. In contrast, DiGA—introduced through the Digital Healthcare Act (“Digitale-Versorgung-Gesetz”, DVG) in 2019—are regulated medical devices requiring approval based on safety, functionality, and efficacy. Their integration into the healthcare system and reimbursement by health insurers enhance credibility and adoption [7]. Currently, two DiGA for smoking cessation, “Smoke Free” and “NichtraucherHelden”, are listed in the official DiGA directory after demonstrating positive health outcomes [8]. Being listed means they are approved for reimbursement by statutory health insurance in Germany [7]. In this study, “digital smoking cessation interventions” (DSCIs) refer to both freely available and prescription-based apps that support long-term smoking cessation through features such as savings tracking, motivational support, craving management, and health improvement insights.

Studies show DSCIs can effectively reduce problematic smoking behavior through targeted content and communication strategies [9–11]. Data from the “NichtraucherHelden” app indicate significantly higher abstinence rates compared to standard care [12]. Given their relevance, large-scale surveys in Germany—such as the Study on Tobacco Use (“Deutsche Befragung zum Rauchverhalten”; DEBRA), funded by health ministries since 2016 [13] and the Smoking Cessation Study (“Rauchstopp-Studie”; RauS) by the Institute for Addiction Research in Frankfurt [14]—have included initial questions on DSCI use. However, comprehensive research on their adoption in Germany remains limited. To better understand the potential of these interventions, it is not only their effectiveness that matters but also whether people are actually willing to use them. While DSCIs provide a wide range of supportive functions, their mere availability does not guarantee actual uptake. Ultimately, the willingness of individuals to engage with such tools determines their potential impact. Understanding user acceptance, defined as the individual’s readiness to use a technological system [15], is therefore essential to identify barriers to adoption and to inform strategies for improving uptake.

In technology research, acceptance has been examined for many years through theoretical frameworks. One of the most established and widely applied models is the Unified Theory of Acceptance and Use of Technology 2 (UTAUT2). This model consolidates various determinants of technology adoption and has been successfully applied in multiple digital health contexts [16–19]. It therefore provides a suitable framework to empirically examine the acceptance of DSCIs in Germany. The aim of this study is to investigate the acceptance of DSCIs among a sample of smokers in Germany and to identify key determinants influencing their intention to use these interventions, including factors related to prescription, reimbursement, and user characteristics.

## Materials and methods

This study was designed as a cross-sectional online survey to investigate the acceptance of DSCIs. The survey targeted current smokers, occasional smokers, and former smokers to capture a broad range of user perspectives. Descriptive statistics, bivariate analyses, and logistic regression models were used to examine predictors of acceptance.

### Inclusion criteria

Eligibility criteria were:

- age ≥ 18 years,
- current or former tobacco use,
- residency in Germany and,
- inclusion of both DSCI users and nonusers.

### Questionnaire

The questionnaire comprised five sections: smoking behavior, DSCI experience, acceptance factors, DSCI acceptance, and sociodemographic data.

For the smoking behavior domain, we relied on items from the large German population-based DEBRA study, which provide validated and widely accepted measures of tobacco use in Germany [20].

Acceptance and its associated factors were based on the UTAUT2 framework, with items specifically measuring the core constructs: Performance Expectancy (PE), Effort Expectancy (EE), Facilitating Conditions (FC) and Social Influence (SI) [21]. In addition, the survey included items developed to capture additional factors relevant to digital health applications and smoking cessation. These factors were selected based on previous studies on the acceptance of digital health applications and included Willingness to Pay (WP), Perceived Trust (PT) [18, 22], Self-Efficacy (SE) [23], Perceived Disease Threat (PDT) [18, 22, 24], as well as regulatory aspects such as DiGA Status (DS) [16, 25] and Data Protection (DP) [16, 25]. Minor modifications were made to the items to tailor them to the smoking cessation context while preserving their conceptual validity.

Where validated German versions were available, these were applied directly. For items without existing translations, a forward translation was performed in accordance with established methodological principles. The wording was subsequently reviewed and refined within the research team to ensure linguistic clarity and conceptual consistency.

Each item is rated on a five-point Likert scale from 1 (strongly disagree) to 5 (strongly agree). The scale enables differentiated responses, including a neutral midpoint, and included a “no answer” option (supplementary material 1 and 2).

To ensure clarity, comprehensibility, and technical functionality, the survey was pretested with eight participants. Although a full psychometric validation in this specific population has not yet been conducted. Participation was anonymous, voluntary, and required informed consent in accordance with the General Data Protection Regulation [26]. Ethical approval was obtained from the Ethics Committee of the University of Applied Sciences Zwickau.

To encourage participation, respondents could—voluntarily and independently of the survey—enter a prize draw for one of five gift vouchers [27].

### Recruitment

The online survey was conducted via LimeSurvey between April and July 2024. Recruitment was primarily done through social media.

Initially, Instagram accounts related to smoking, each with at least 300 followers were identified, resulting in a total of twelve pages. Followers of these accounts were contacted daily via direct messages with a personalized explanation of the study and an invitation to participate. In collaboration with the German Federal Center for Health Education (“Bundeszentrale für gesundheitliche Aufklärung”, BZgA), the survey was promoted via the Instagram page “rauchfrei_info”.

In addition, Facebook groups focused on smoking, each with over 4,000 members, were identified and contacted to request posting the survey link, with one group agreeing to share it.

Recruitment was also supported through certified smoking cessation counselors. Using BZgA and German Cancer Research Center (“Deutsches Krebsforschungszentrum”, DKFZ) database, a total of 45 counselors were contacted. Of these, five assisted in distributing the survey via posters, group counselling sessions, and email list.

### Statistical analysis

All statistical analyses were conducted using IBM SPSS Statistics Version 29 (α = 0.05, two-tailed). After data cleaning, variables were first examined descriptively. Frequency tables were used for nominal and ordinal variables, while means, standard deviations, and 95% confidence intervals were reported for metric variables.

Chi-square tests and cross-tabulations examined univariate associations between categorical variables. Group differences between DSCI acceptance and non-acceptance were tested using the nonparametric Mann–Whitney U test. Correlation analyses were conducted depending on measurement levels to prepare for regression.

To address the main research question, a binary logistic regression was performed with DSCI acceptance (1 = yes; 0 = no) as the dependent variable. Acceptance was assessed using three items on a five-point Likert scale, with higher values indicating greater acceptance. Participants were classified as “acceptance” if their total score across the three items exceeded the rounded sum of the item means (Item 1: M = 3.74, Item 2: M = 3.37, Item 3: M = 3.24), which required a consistently positive response (agreement) on all three items, and as “non-acceptance” otherwise.

The regression included sex, age, smoking duration, PE, SE, WP, PDT, PT, and DS as predictors. The analysis used the inclusion method (Wald) and only fully completed questionnaires were considered; incomplete cases were excluded through listwise deletion. No imputation procedures were applied, as missing values on the predictors were assessed globally using the MVA procedure with Little’s test [28]. The correlations were not statistically significant. To ensure sufficient observations per response category for basic statistical analyses, items for each independent variable were combined to create a composite score [29]. SPSS was used to calculate cut-off values to divide participants into three approximately equal groups, which were categorized as low, medium, or high.

Model assumptions were tested using variance inflation factors (VIF < 5) for multicollinearity. Model fit was assessed via likelihood ratio test, Nagelkerke R-Squared, the omnibus test, and classification tables. Odds ratios (OR) quantified effect sizes.

## Results

This section presents the key findings of the study, including sample characteristics, DSCI usage, acceptance levels, and factors influencing acceptance.

### Study sample

**Fig. 1:**
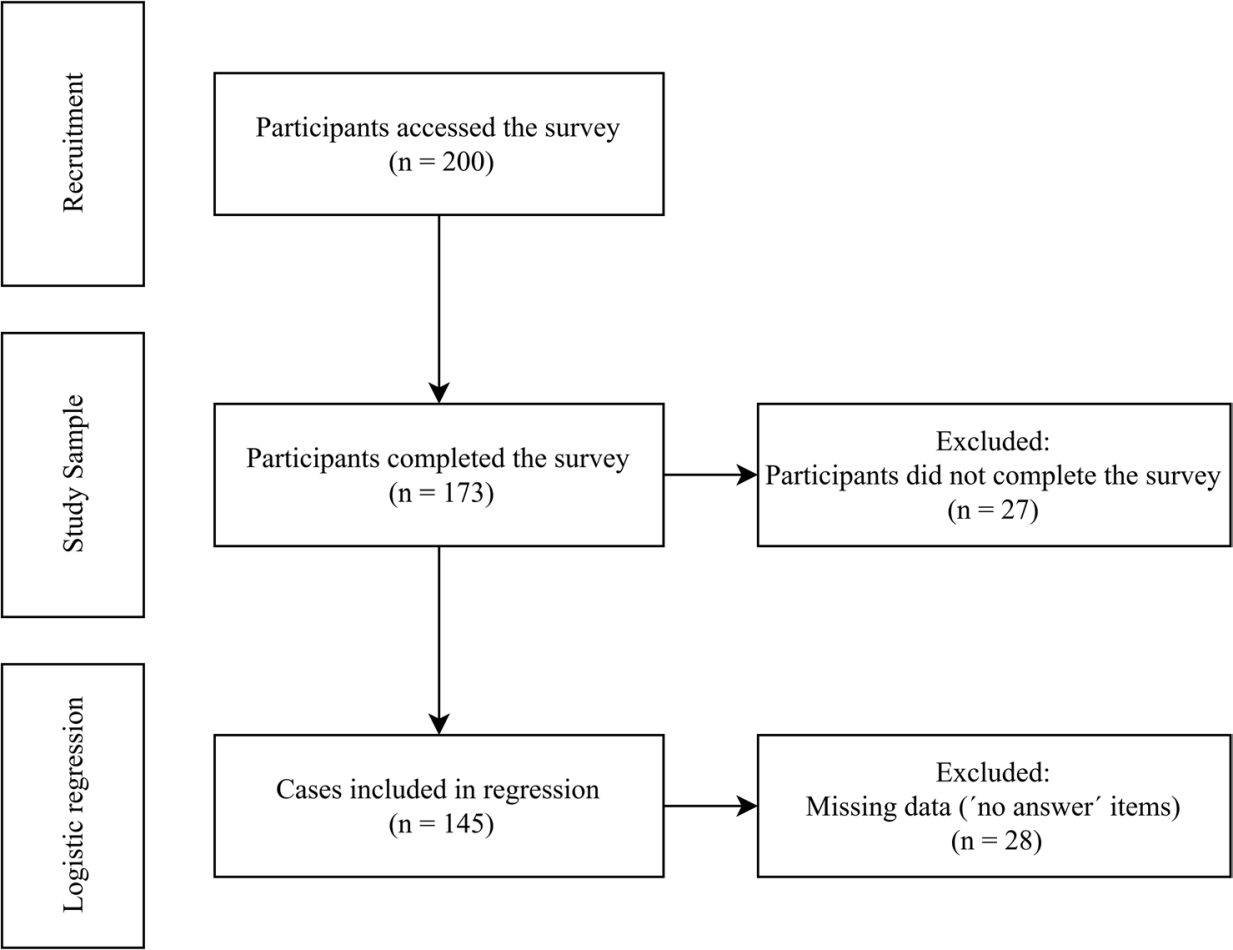
Flow diagram of the survey participation and analysis

A total of 200 participants accessed the survey, 173 of whom completed it fully (Fig. 1). The sample consisted predominantly of smokers (101/173; 58.38%), with women comprising 61.85% (107/173) of participants (Table 1). The average age was 35.28 years (SD = 12.43). Most participants held a general university entrance qualification (84/173; 48.55%) or a secondary school diploma (53/173; 30.64%), and lived primarily in large cities (62/173; 35.84%) or rural areas (38/173; 21.97%).

**Table 1:** Sociodemographic characteristics of smokers, occasional smokers, and former smokers (*N* = 173), n (%)

	Smokers 101 (58.38)	Occasional Smokers 7 (4.05)	Former Smokers 65 (37.57)	Total 173 (100)
Gender				
Male	41 (40.59)	3 (42.86)	21 (32.31)	65 (37.57)
Female	59 (58.42)	4 (57.14)	44 (67.69)	107 (61.85)
Diverse	1 (0.99)	0 (0.00)	0 (0.00)	1 (0.58)
Age				
18–24 years	26 (25.74)	5 (71.43)	2 (3.08)	33 (19.08)
25–39 years	50 (49.50)	2 (28.57)	35 (53.85)	87 (50.29)
40–64 years	25 (24.75)	0 (0.00)	23 (35.38)	48 (27.75)
65 years or older	0 (0.00)	0 (0.00)	5 (7.69)	5 (2.89)
Education				
No secondary education	4 (3.96)	0 (0.00)	3 (4.62)	7 (4.05)
Secondary education	37 (36.63)	2 (28.57)	14 (21.54)	53 (30.64)
Higher secondary education	16 (15.84)	1 (14.29)	12 (18.46)	29 (16.76)
University entrance qualification	44 (43.56)	4 (57.14)	36 (55.38)	84 (48.55)
Residence				
Rural areas	27 (26.73)	0 (0.00)	11 (16.92)	38 (21.97)
Small towns	24 (23.76)	2 (28.57)	10 (15.38)	36 (20.81)
Medium-sized towns	15 (14.85)	2 (28.57)	20 (30.77)	37 (21.39)
Large cities	35 (34.65)	3 (42.86)	24 (36.92)	62 (35.84)

*N* Total sample size, *n* Subsample size

Participants reported a mean smoking initiation age of 17.06 years (SD = 5.33) and an average of 3.42 previous quit attempts (SD = 4.96). The overall mean smoking duration was 18.21 years (SD = 12.50), with significant differences between smoking status groups (current smokers: M = 17.09, SD = 11.34; occasional smokers: M = 4.86, SD = 2.85; former smokers: M = 21.42, SD = 13.66) (Table 2).

**Table 2:** Smoking behavior among smokers, occasional smokers, and former smokers (*N* = 173)

	Smokers *n* = 101 (58.38%)	Occasional Smokers *n* = 7 (4.05%)	Former Smokers *n* = 65 (37.57%)	*p* value*	Total *N* = 173
Smoking duration (years)					
M	17.09	4.86	21.42	**0.005**	18.21
SD	11.34	2.85	13.66		12.50
95% CI	14.85–19.33	2.22–7.50	18.03–24.78		16.34–20.10
Quit attempts (number)					
M	3.34	2.43	3.65	0.553	3.42
SD	5.82	1.13	3.58		4.96
95% CI	2.19–4.49	1.38–3.48	2.76–4.53		2.67–4.16
Motivation to quit (number)					
M	6.00	6.00	n. a.	0.914	6.00
SD	3.00	3.00	n. a.		3.00
95% CI	5.00–7.00	3.00–8.00	n. a.		5.00–7.00

*CI* Confidence interval, *M* Mean, *N* Total sample size, *n* Subsample size, *n. a.* Not applicable, *SD* Standard deviation

*Chi-square test

### Usage behavior

Of the total sample, 41.62% (72/173) reported having used a DSCI, with the majority of users being female (79.17%; 57/72; *p* < 0.001). On average, users were 37.25 years (SD = 10.42) old. The most frequently used interventions were “Smoke Free” (44.44%; 32/72) and “NichtraucherHelden” (25.00%; 18/72), with most users reporting an average usage duration of one to three months. A significant association was found between smoking status and DSCI use (*p* < 0.001) (Table 3). Among those who quit smoking and used a DSCI, 56.41% (22/39) stated they were able to quit successfully with the help of the intervention.

**Table 3:** Association between smoking status and use of DSCI (*N* = 173), n (%)

	Non-User 101 (58.38)	User 72 (41.62)	*p* value*	Total 173 (100)
Smokers	68 (67.33)	33 (45.83)	**< 0.001**	101 (58.38)
Occasional Smokers	7 (6.93)	0 (0.00)		7 (4.05)
Former Smokers	26 (25.74)	39 (54.17)		65 (37.57)

*N* Total sample size, *n* Subsample size

*Chi-square test

### Acceptance levels

**Fig. 2:**
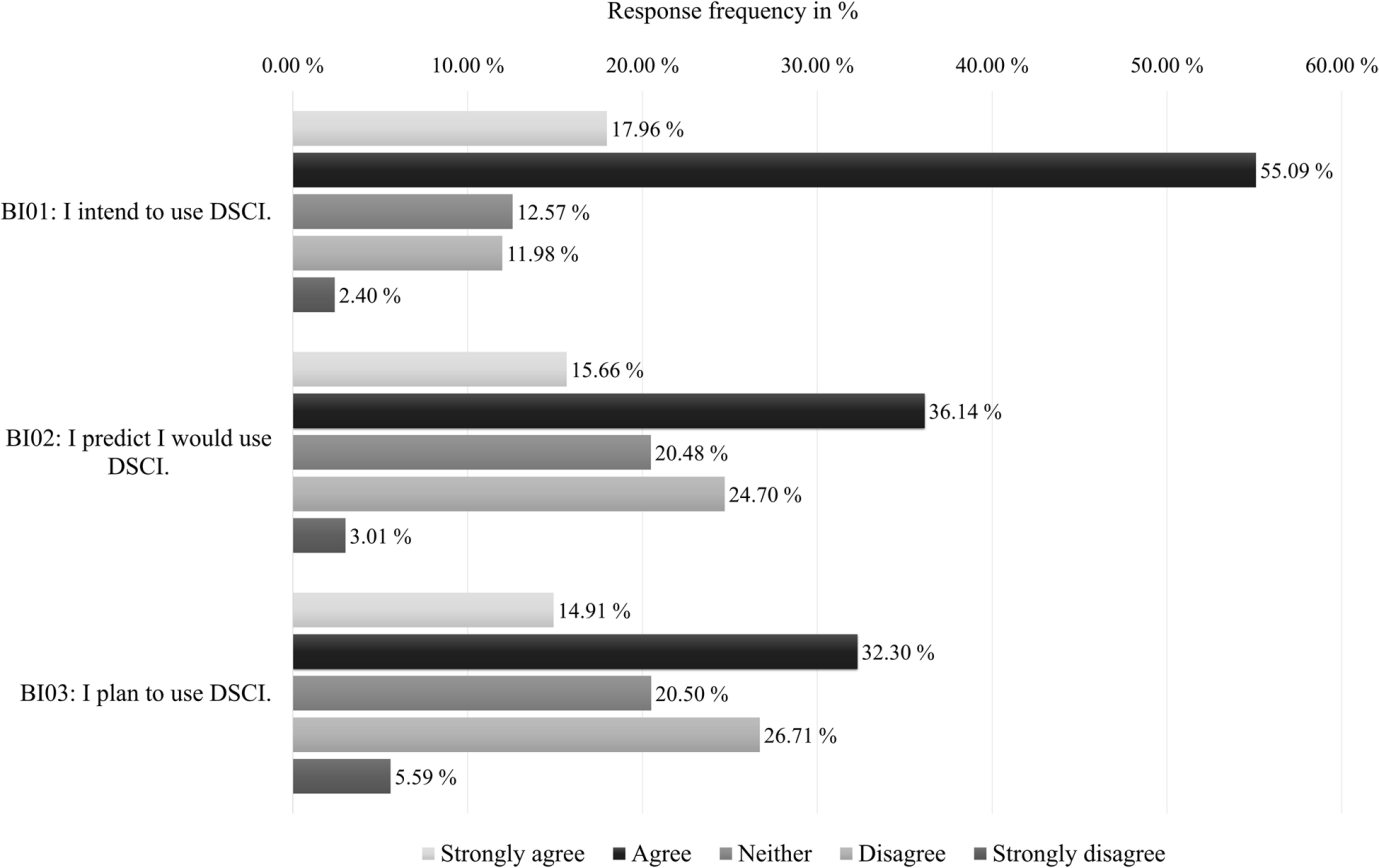
Participants’ behavioral intentions to use the DSCI, based on agreement levels with three acceptance-related items (response category “no answer” was excluded from analysis)

Participants generally showed a high intention to use the DSCI, with 73.05% (122/167; 6/173 “no answer”) agreeing they could envision using it (again) (in case of relapse), although only 47.20% (76/161; 12/173 “no answer”) plans to use the DSCI regularly (Fig. 2).

### Factors influencing acceptance

Most participants (168/171; 98.24%; 2/173 “no answer”) had the necessary technical equipment and knowledge to use DSCIs. However, only 29.00% (49/169; 4/173 “no answer”) were willing to pay for DSCI usage. A high awareness of smoking-related health risks was evident, with 98.82% (168/170; 3/173 “no answer”) recognizing the harmful effects of smoking and 81.98% (141/172; 1/173 “no answer”) expressing concern about their own smoking behavior. Moreover, 83.34% (140/168; 5/173 “no answer”) of participants indicated they would be more inclined to use a DSCI if its efficacy were scientifically validated. Data protection concerns were less significant, with only 24.12% (41/170; 3/173 “no answer”) expressing concerns about personal data protection when DSCI was used (supplementary material 3).

### Logistic regression analysis

The logistic regression included predictors that showed significant associations with DSCI acceptance in the Mann–Whitney U tests, namely gender, age, smoking duration, PE, SE, WP, PDT, PT, and DS. Variables that did not demonstrate significant correlations, such as previous quit attempts, place of residence, educational level, EE, FC, and SI were excluded from the model. The analysis was conducted on 145 complete cases, after excluding 28 incomplete responses due to missing data resulting from the “no answer” response option (Fig. 1). In our sample, SE was identified as a strong predictor, with individuals exhibiting high self-efficacy demonstrating 3.829 times greater odds of accepting DSCIs (95% CI: 1.263–11.609; *p* = 0.018). Additionally, WP significantly influenced acceptance, as participants willing to pay showed 3.760 times higher odds of DSCI acceptance (95% CI: 1.329–10.634; *p* = 0.013). Perceived importance of physician prescription and scientific evidence (DS) was also associated with increased odds of acceptance, with participants valuing these aspects showing 3.850 times greater odds (95% CI: 1.248–11.873; *p* = 0.019) (Table 4). The regression model showed a Nagelkerke R-Square of 0.377. The Hosmer–Lemeshow test (χ² (8) = 9.363, *p* = 0.313) indicated an adequate model fit, and the Omnibus test confirmed overall model significance. The model’s prediction accuracy was 73.60%.

**Table 4:** Logistic regression results for acceptance of the DSCI (*N* = 173; complete cases for regression *n* = 145)

	Acceptance		
	*p* value*	OR	95% CI
Age			
18–24 years	Reference		
25–39 years	0.629	1.418	0.345–5.827
40–64 years	0.822	1.258	0.171–9.252
65 years or older	0.735	1.833	0.055–61.103
Gender			
Male	Reference		
Female	0.655	0.815	0.333–1.998
Diverse	> 0.999	> 0.001	0.000–n.a.
Smoking duration			
1–7 years	Reference		
8–12 years	0.138	0.337	0.080–1.417
13–19 years	0.683	0.727	0.157–3.366
20–28 years	0.378	0.467	0.086–2.540
29–54 years	0.571	1.886	0.210–16.965
PE			
Low	Reference		
Middle	0.459	1.422	0.560–3.613
High	0.349	1.644	0.581–4.653
SE			
Low	Reference		
Middle	0.061	2.951	0.952–9.149
High	**0.018**	3.829	1.263–11.609
WP			
Low	Reference		
Middle	0.759	1.205	0.366–3.972
High	**0.013**	3.760	1.329–10.634
PDT			
Low	Reference		
Middle	0.516	1.444	0.477–4.370
High	0.471	1.508	0.493–4.612
PT			
Low	Reference		
Middle	0.974	0.983	0.347–2.784
High	0.073	2.745	0.909–8.288
DS			
Low	Reference		
Middle	0.991	1.007	0.326–3.108
High	**0.019**	3.850	1.248–11.873

*DS* DiGA-Status, *n* Subsample size, *n. a*. Not applicable, *OR* Odds Ratio, *PDT* Perceived Disease Threat, *PE* Performance Expectancy, *PT* Perceived Trust, *SE* Self-Efficacy *WP* Willingness to Pay

*Chi-square test

## Discussion

This study addressed the limited research on DSCI adoption in Germany by examining user behavior, acceptance levels, and influencing factors—including the role of prescription and reimbursement. In our sample, participants reported a generally high intention to use DSCIs, particularly among former smokers and women. Key predictors of acceptance in our survey were SE, WP, and DS.

### Usage behavior

In our sample, 41.62% (72/173) of participants reported having used a digital DSCI, compared to 2.20% in the German DEBRA study [13]. Similarly, a study conducted within the “European Regulatory Science on Tobacco: Policy implementation to reduce lung diseases” Project (EUREST-PLUS) reported low use of cessation support across eight European countries, with somewhat higher uptake in England, where substantial investment in tobacco control and digital programs has taken place [30]. However, even in England, only 2.70% of smokers reported using any form of digital cessation support, and just 0.60% had ever used a dedicated app [31]. While these comparisons provide useful context, differences in study design and sample composition limit comparability. The higher usage rate in our study may reflect the recruitment of more motivated smokers who were open to digital health solutions.

The average age of DSCI users in our sample was 37 years, which is younger than the 47 years reported for DiGA users by the German Health Insurance Association in 2024 [32] and the 46 years reported by “NichtraucherHelden” [12]. Similar age patterns were found in the United States, where most cessation interventions users were between 35 and 54 years [33].

This may indicate that older smokers, who are more likely to have smoking-related health conditions and regular medical contact, tend to use physician-prescribed, reimbursed interventions, whereas younger individuals prefer freely available digital solutions [34].

The high proportion of female DSCI users (79.17%; 57/72) in our sample aligns with prior findings in Germany, where 69.00% of “NichtraucherHelden” [35] and 73.00% of DiGA users were female [32]. Similar patterns have been reported in the United States and Norway [33, 36]. The EUREST-PLUS project likewise found that in England, women were more likely than men to use smoking cessation medication, whereas in the Netherlands, men more often received quitting advice from health professionals [30].

The lower uptake among men may reflect established gender differences in health behavior: men often display greater risk tolerance, seek professional help less frequently [37], and may perceive quitting without external assistance as a sign of autonomy or strength [38, 39]. Within the scope of our findings, targeted design strategies could include gamified elements or motivational feedback to strengthen men’s engagement.

The higher DSCI uptake among more educated and urban participants in our sample is consistent with findings from the EUREST-PLUS project and a study on users of the “ExSmokers iCoach” app, a free cessation tool developed by BrandNewHealth for the European Commission’s “Ex-Smokers Are Unstoppable” campaign [40]. These results suggest that digital literacy and health awareness may facilitate DSCI adoption. To reduce access disparities, future interventions should consider strategies to engage individuals with lower education levels, for example through simplified design or integration into primary care.

The observed usage patterns should be interpreted in light of the recruitment strategy via social media. The visibility of posts on platforms like Instagram is influenced by algorithmic mechanisms, which can determine who sees and engages with content. Certain strategies, such as engagement bait, may reduce post visibility [41–43]. Consequently, the observed age, gender, and engagement patterns may partly reflect platform dynamics rather than only individual characteristics. As a result, participation and engagement may reflect platform-specific visibility mechanisms as much as individual characteristics, potentially reinforcing selection bias within the sample.

### Acceptance levels and influencing factors

The average acceptance score of 3.45 in our sample aligns with those for blood pressure (M = 3.26) [24] and multiple sclerosis interventions (M = 3.11) [19], suggesting a positive perception among respondents.

WP for DSCI in our data was low (29.00%; 49/169), consistent with previous findings that only 27.00% of Germans would cover DiGA costs out of pocket [16]. Similarly, a study in the United Kingdom found that users were willing to pay only modest amounts (up to £7) for high-quality DSCI [44]. In contrast, data from German statutory health insurance show substantially higher uptake when interventions are prescribed and reimbursed [45, 46]. Moreover, findings from a German study indicate that willingness to pay tends to increase with prior experience [16]. The findings may suggest that reducing financial barriers and integrating DSCI into reimbursement pathways could help facilitate their wider acceptance and use.

SE was a significant predictor of DSCI acceptance in our sample, consistent with behavioral theories emphasizing the role of confidence and perceived control in technology use [16, 17]. Moreover, findings from the “ExSmokers iCoach” study also suggests that intensive and sustained use of such interventions can strengthen users’ SE over time [40]. To enhance DSCI adoption, interventions could incorporate elements that strengthen self-efficacy, such as clear guidance, positive feedback, and gradual goal setting [47].

Privacy concerns were not a significant factor, despite Germany’s strict data protection regulations. This finding is consistent with research showing that privacy concerns represent only moderate barriers to digital health adoption [16, 22, 25, 48]. Participants may perceive smoking cessation data as relatively low risk or consider the potential health benefits to outweigh privacy concerns. Alternatively, some may consciously accept data-sharing trade-offs to access support. Future studies should further examine these motivations to better understand privacy-related decision-making in digital health contexts.

### Role of prescription and reimbursement status

In a German study, 53.00% of respondents reported they would only use a digital intervention if prescribed by a physician and scientifically proven effective [16]. Similarly, 83.34% (140/168) of this study’s participants stated they would be more inclined to use a DSCI if its efficacy were scientifically validated. Comparable results were reported in the United Kingdom, where participants preferred interventions developed by trusted healthcare organizations rather than hypothetical commercial providers [44].

Therefore, DSCIs could be integrated into general practice and addiction care to complement counseling or pharmacotherapy. Potential barriers include limited consultation time, insufficient training, reimbursement uncertainties, and data privacy concerns. Addressing these could support broader implementation in routine care.

### Limitations

This study has several limitations that should be considered when interpreting the results. First, the overall sample size was modest, limiting statistical power and the ability to generalize findings to the wider smoking population. However, studies with similar sample sizes are occasionally seen in smoking cessation research [49]. Second, recruitment via social media and other online channels may have introduced sampling bias, as younger, digitally literate, health-conscious, and socioeconomically advantaged individuals are more likely to participate in such surveys, while older adults, individuals with lower socioeconomic status, or those with limited digital health literacy may be underrepresented [50]. This limited the generalizability of our findings to the broader smoking population in Germany, where digital literacy and health seeking behaviors vary substantially across age and socioeconomic groups. Third, the use of an online survey design carries inherent methodological limitations, including potential misunderstandings of questions and the inability to verify responses. In addition, the response rate could not be determined because the total number of individuals reached through social media was unknown. This introduces a potential nonresponse bias, as individuals with lower interest in digital health or smoking cessation may have been less likely to participate [51]. Consequently, the results may overestimate awareness and acceptance of DSCIs within the general smoking population. Social desirability bias may have influenced participants’ responses, potentially leading to underreporting of smoking behavior and overreporting of positive attitudes toward digital smoking cessation interventions [52]. Finally, as the data were cross-sectional and self-reported, causal relationships cannot be inferred.

### Further research

Future research should refine key determinants of DSCI acceptance, such as SE, DS, WP, PT, PE, and SI. Moreover, the findings suggest that the UTAUT2 model may not fully capture the specific dynamics of the healthcare sector. To better reflect the unique characteristics of digital health interventions, the model could be extended to include additional factors. Trust in healthcare providers and digital platforms may influence perceived usefulness and intention to use DSCIs, while regulatory status, such as DiGA approval, could affect perceived credibility. Data privacy concerns may act as barriers, whereas reimbursement options through statutory health insurance could facilitate adoption. Perceived disease burden and integration into established care pathways may influence engagement and adherence. Health inequalities, including socioeconomic status, education, and digital health literacy, could moderate acceptance across different population groups, and the quality of the physician–patient relationship could serve as an additional predictor, reflecting trust and perceived support. Incorporating these factors would enhance the model’s ability to capture the realities of digital health adoption and improve its predictive power. Future studies should also aim for larger, more diverse samples, potentially supported by targeted recruitment through smoking cessation counsellors (e.g., BZgA, DKFZ, Deutsche Herzstiftung) and general practitioners.

Further research should examine gender-specific usage patterns—particularly why women are more likely to use DSCIs—and explore strategies to better engage male users. Additionally, the influence of prior digital experience, differences between DiGA-certified and freely available DSCIs, user adherence, and long-term effectiveness warrant closer examination.

## Conclusion

This study suggests a potential increase in DSCI adoption compared with previous research. Within our sample, the primary user group consisted of middle-aged women, most of whom relied on certified DiGA for smoking cessation, primarily as a tool to support long-term abstinence.

Although participants expressed generally positive attitudes toward DSCIs, their actual usage rates were lower than their approval levels, indicating only moderate acceptance within this sample. Furthermore, WP, PT, and DS emerged as statistically significant determinants of acceptance in this specific group, but further studies with larger and more diverse populations are needed to validate these results.

## Supplementary Information

supplementary material 1.

supplementary material 2.

supplementary material 3.

## Data Availability

The datasets generated and/or analyzed during the current study are available from the corresponding author upon reasonable request. The English and German versions of the questionnaire are provided as supplementary material.

## Abbreviations

BI: Behavior Intention
BZgA: German Federal Center for Health Education (“Bundeszentrale für gesundheitliche Aufklärung”)
CI: Confidence interval
DEBRA: German Study on Tobacco Use (“Deutsche Befragung zum Rauchverhalten”)
DiGA: German state-certified Digital Health Application (“Digitale Gesundheitsanwendungen”)
DS: DiGA-Status
DKFZ: German Cancer Research Center (“Deutsches Krebsforschungsinstitut”)
DP: Data Protection
DSCI: digital smoking cessation intervention
DVG: Digital Healthcare Act (“Digitale-Versorgung-Gesetz“)
EE: Effort Expectancy
EUREST-PLUS: European Regulatory Science on Tobacco: Policy implementation to reduce lung diseases
FC: Facilitating Conditions
M: Mean
N: Total sample size
n: Subsample size
n. a.: Not applicable
OR: Odds Ratio
PDT: Perceived Disease Threat
PE: Performance Expectancy
PT: Perceived Trust
RauS: German Smoking Cessation Study (“Rauchstopp-Studie”)
SD: Standard deviation
SE: Self-Efficacy
SI: Social Influence
UTAUT: Unified Theory of Acceptance and Use of Technology
WP: Willingness to Pay
VIF: Variance inflation factors
