# Supplementary material for: Understanding acceptance of digital smoking cessation interventions: user behavior, key influencing factors, and the role of reimbursement
Source: BMC Public Health. 2025 Dec 12;26:218. doi: 10.1186/s12889-025-25472-4 (PMC12817400; doi:10.1186/s12889-025-25472-4)
Supplement: Supplementary file 1 — supplementary material 1. [file 12889_2025_25472_MOESM1_ESM.docx]

1. **Questionnaire – German Version (original)**

**Teil1: Angaben zum Rauchverhalten (1 von 8)**

**Bitte beachten Sie, dass sich die folgenden Fragen auf Tabakzigaretten und nicht auf elektronische Zigaretten beziehen.**

| Nr. | Codierung | Variable | Frage | Antwortmöglichkeiten | Skalenniveau | Quelle |
| --- | --- | --- | --- | --- | --- | --- |
| 01 | sok | Rauchen | Welche der folgenden Aussagen trifft auf Sie am ehesten zu? | Single Choice   1. Ich rauche täglich. 2. Ich rauche nicht jeden Tag, aber mindestens 1 Packung Zigaretten pro Monat. 3. Ich rauche nur zu bestimmten Gelegenheiten (z.B. gesellschaftliche Anlässe). 4. Ich habe im letzten Jahr vollständig mit dem Rauchen aufgehört. 5. Ich habe vor mehr als einem Jahr vollständig mit dem Rauchen aufgehört. 6. Ich habe noch nie geraucht. -> Umfrage beendet, Nutzung von Quoten | Nominal | Aus Baseline-Fragebogen der DEBRA Studie [1]  Rahmen der Umfrage:   - Konventionelle Tabakzigaretten - Raucher (1) und (2) - Gelegenheitsraucher (3) - Ex-Raucher (4) und (5) - Nichtraucher (6) |
| 02 | tr01 | Rauchbeginn | In welchem Jahr haben Sie erstmalig mit dem Rauchen begonnen?  1920<tr01<2024  Keine Pflichtangabe | Numeric Input | Metrisch | Aus Baseline-Fragebogen der DEBRA Studie [1]  BisQuits Studie fragt nach Alter bei Rauchbeginn [2] |
|  | tr02 | Rauchende | [If answered 01 with (5)] In welchem Jahr haben Sie vollständig mit dem Rauchen aufgehört?  tr02<tr02<2023  keine Pflichtangabe | Numeric Input | metrisch | Aus Fragen Aktivrauchen – Kurzversion (Erwachsene) von [3] |
| 03 | aw | Aufhörwunsch | [if answered 01 with (1) OR (2) OR (3)] Wie stark ist aktuell Ihr Wunsch mit dem Rauchen aufzuhören? | Überhaupt nicht stark (0)  Extrem stark (10)  Visuelle Analogskala von 0 bis 10 | Ordinal | Aus BisQuits T0 Fragebogen von Rupp A, Blank J, Ehmann M et al. (2014) Rupp, Blank, Ehmann, Pousset, Mühlig, Sehl, Fuchs, Rüther, Linhardt, Grah, Kreuter[2] |
| 04 | av01 | Rauchstoppversuche | [if answered 01 with (1) OR (2) OR (3)] Wie viele **ernstgemeinte** Versuche haben Sie bislang insgesamt unternommen, mit dem Rauchen aufzuhören?  av01>0 | Numeric Input | Metrisch | BisQuits T0 Fragebogen von Rupp A, Blank J, Ehmann M et al. (2014) [2] |
|  | av02 |  | [if answered 01 with (4) OR (5)] Wie viele **ernstgemeinte** Versuche haben Sie insgesamt unternommen, mit dem Rauchen aufzuhören?  avo2>1 | Numeric Input | Metrisch | BisQuits T0 Fragebogen von Rupp A, Blank J, Ehmann M et al. (2014) [2] |

**Teil 2: Erfahrungen mit digitalen Gesundheitsinterventionen zur Rauchentwöhnung (2 von 8)**

Digitale Rauchentwöhnungsinterventionen (DRI) sind mobile Apps, die Rauchenden helfen sollen, ihre Tabakabhängigkeit zu überwinden und dauerhaft mit dem Rauchen aufzuhören (z. B. SmokeFree – Rauchen aufhören, QuitNow!). Typische Funktionen umfassen ein Rauchstopp-Programm, Motivationshilfen, Ablenkungsmöglichkeiten bei Rauchverlangen und Informationen zum Gesundheitszustand.

| Nr. | Codierung | Variable | Frage | Antwort | Skala | Quelle |
| --- | --- | --- | --- | --- | --- | --- |
| 05 | exp | Erfahrung | Haben Sie jemals eine digitale Rauchentwöhnungsintervention genutzt? | Single Choice   1. Ja 2. Nein | nominal | New Item |
| 06 | app | Digitale Gesundheitsintervention | [If answers 05 with (1)]: Welche digitale(n) Rauchentwöhnungsintervention(en) haben Sie genutzt? | Multiple Choice   1. NichtraucherHelden-App 2. SmokeFree 3. Rauchen aufhören – Kwit (iOs)/Kwit – Rauchen war gestern (Android) 4. QuitNow! 5. Rauchen aufhören – Easy Quit 6. Rauchfrei Lite (iOs)/Rauchfrei – aufhören zu rauchen (Android) 7. Rauchen aufhören mit Flamy 8. QuitSure: Rauchen aufhören 9. TK-RauchFrei 10. Sonstige | nominal | Auswahl orientiert sich an den Downloadzahlen und an der Anzahl der Rezensionen im Google Play Store und Apple App Store, siehe Kapitel  ACHTUNG! Bezeichnung unterscheiden sich in den Stores |
| 07 | tn | Nutzungsdauer | [If answered 05 with (1)]: Wie lange haben Sie die digitale Rauchentwöhnungsintervention genutzt?  Hinweis: Wenn Sie mehrere Interventionen genutzt haben, beantworten Sie bitte die Frage bezüglich der Anwendung, die Sie am längsten verwendet haben. | Single Choice   1. bis 3 Tage 2. über 3 Tage bis 1 Woche 3. über 1 Woche bis 1 Monat 4. über 1 Monat bis 3 Monate 5. über 3 Monate bis 6 Monate 6. über 6 Monate bis 12 Monate 7. länger als 12 Monate | metrisch | Frage stammt aus der Studie von Uncovska M, Freitag B, Meister S et al. (2023) [4]  Antwortkategorien orientieren sich an der Anwendungsdauer von SmokeFree und der NichtraucherHelden-App |
| 08 | rf01 | Rauchfrei | [If answered 01 with (1) OR (2) OR (3) AND 05 with (1)] Wie viele Monate waren Sie nach Ende der Nutzung der digitalen Rauchentwöhnungsintervention rauchfrei?  Hinweis:   - Wenn Sie weniger als 1 Monat rauchfrei waren, tragen Sie bitte eine 0 ein. - Wenn Sie mehrere Interventionen genutzt haben, beantworten Sie bitte die Fragen bezüglich der Anwendung, die Sie am längsten verwendet haben.   Keine Pflichtangabe | Numeric Input, ganzzahlig, 2 Stellen  _ Monate | Metrisch | Orientiert sich an BISQUITS T0 Fragebogen von Rupp, Blank, Ehmann, Pousset, Mühlig, Sehl, Fuchs, Rüther, Linhardt, Grah, KreuterRupp A, Blank J, Ehmann M et al. (2014) [2] |
|  | rf02 | Rauchfrei | [If answered 1 with (4) OR (5) AND 05 with (1)] Haben Sie mit Hilfe einer digitalen Rauchentwöhnungsintervention nachhaltig mit dem Rauchen aufgehört? | Single Choice   1. Ja 2. nein | nominal | NEW ITEM |

Single-choice, 5-stufige Likert Skala, bestehend aus den Optionen "Stimme überhaupt nicht zu“(1), "Stimme nicht zu“(2), "Weder noch“(3), "Stimme zu“(4), "Stimme voll und ganz zu“(5), ergänzt durch die Möglichkeit "keine Antwort“(0) -> Skala ordinal

Bitte beachten Sie, dass die Formulierung der folgenden Aussagen bewusst so gestaltet wurde, dass sie von Rauchenden sowie ehemaligen Rauchenden, mit oder ohne vorherige Nutzungserfahrung gleichermaßen beantwortet werden können.

| Nr. | Codierung | Variable | Aussage | Skala | Quelle |
| --- | --- | --- | --- | --- | --- |
| Bitte geben Sie an, inwiefern Sie den folgenden Aussagen bezüglich Ihrer Nutzererfahrungen mit digitalen Rauchentwöhnungsinterventionen zustimmen oder nicht zustimmen.  Nachfolgend lesen Sie eine Reihe von Aussagen. Wenn Sie einer Aussage voll und ganz zustimmen, wählen Sie bitte die vorletzte Auswahloption. Wenn Sie der Aussage überhaupt nicht zustimmen, wählen Sie bitte die erste Auswahloption. Mit den Auswahloptionen dazwischen können Sie Ihre Beurteilung abstufen. Ihre Einschätzung ist für die Forschungsarbeit von entscheidender Bedeutung. Falls Sie jedoch eine Aussage nicht beurteilen können oder wollen, wählen Sie die letzte Auswahloption aus.  DRI = digitale Rauchentwöhnungsinterventionen | | | | | |
| 09 | UEX01 | Nutzererfahrungen | DRI helfen, mit dem Rauchen aufzuhören. | ordinal | Uncovska M, Freitag B, Meister S et al. (2023) [4] |
|  | UEX02 |  | DRI helfen beim Umgang mit Rauchgelüsten. | ordinal | Uncovska M, Freitag B, Meister S et al. (2023) [4]  Breil B, Kremer L, Hennemann S et al. (2019) [5], Hennemann S, Beutel ME, Zwerenz R (2016) [6] |
|  | UEX03 |  | DRI verbessern das eigene gesundheitliche Wohlbefinden. | ordinal | Apolinário-Hagen J, Menzel M, Hennemann S et al. (2018) [7] |
|  | UEX04 |  | DRI sind in der Bedienung einfach und verständlich. | ordinal | Schretzlmaier P, Hecker A, Ammenwerth E (2022) [8], Hennemann S, beutel ME, Zwerenz R (2016) [6], Zhang Y, Liu C, Luo S et al. (2019) [9] |
|  | UEX04 |  | DRI sind in der Nutzung einfach zu erlernen. | ordinal | Schretzmaier P, Hecker A, Ammenwerth E (2022) [8], Hennemann S, Beutel ME, Zwerenz R (2016) [6], Zhang Y, Liu C, Luo S et al. (2019) [9] |

**Teil 3: Einflussfaktoren auf die Akzeptanz (3,4,5,6 von 8)**

Single-choice, 5-stufige Likert Skala, bestehend aus den Optionen "Stimme überhaupt nicht zu“(1), "Stimme nicht zu“(2), "Weder noch“(3), "Stimme zu“(4), "Stimme voll und ganz zu“(5), ergänzt durch die Möglichkeit "keine Antwort“(0) -> Skala ordinal

| Nr. | Codierung | Variable | Aussage | Skala | Quelle |
| --- | --- | --- | --- | --- | --- |
| Bitte geben Sie an, inwiefern Sie den folgenden Aussagen bezüglich Ihrer Nutzererwartung zustimmen oder nicht zustimmen.  Nachfolgend lesen Sie eine Reihe von Aussagen. Wenn Sie einer Aussage voll und ganz zustimmen, wählen Sie bitte die vorletzte Auswahloption. Wenn Sie der Aussage überhaupt nicht zustimmen, wählen Sie bitte die erste Auswahloption. Mit den Auswahloptionen dazwischen können Sie Ihre Beurteilung abstufen. Ihre Einschätzung ist für die Forschungsarbeit von entscheidender Bedeutung. Falls Sie jedoch eine Aussage nicht beurteilen können oder wollen, wählen Sie die letzte Auswahloption aus. | | | | | |
| Ich erwarte, dass digitale Rauchentwöhnungsinterventionen… | | | | | |
| 09 | PE01 | Performance Expectancy/Leistungserwartung | … helfen, mit dem Rauchen aufzuhören. | ordinal | Uncovska M, Freitag B, Meister S et al. (2023)[4] |
|  | PE02 |  | … beim Umgang mit Rauchgelüsten helfen. | ordinal | Uncovska M, Freitag B, Meister S et al . (2023) [4]  Breil B, Kremer L, Hennemann S et al. (2019) [5], Hennemann S, Beutel ME, Zwerenz R (2016) [6] |
|  | PE03 |  | …das eigene gesundheitliche Wohlbefinden verbessern. | ordinal | Apolinário-Hagen J, Menzel M, Hennemann S et al. (2018) [7] |
| 10 | EE01 | Effort Expectancy/Aufwandserwartung | …in der Bedienung einfach und verständlich sind. | ordinal | Schretzlmaier P, Hecker A, Ammenwerth E (2022) [8], Hennemann S, Beutel ME, Zwerenz R (2016) [6], Zhang Y, Liu C, Luo S et al. (2019) [9] |
|  | EE02 |  | …in der Nutzung einfach zu erlernen sind. | ordinal | Schretzmaier P, Hecker A, Ammenwerth E (2022) [8], Hennemann S, Beutel ME, Zwerenz R (2016) [6], Zhang Y, Liu C, Luo S et al. (2019) [9] |
| Bitte geben Sie an, inwiefern Sie den folgenden Aussagen bezüglich Ihrer Nutzungsvoraussetzungen zustimmen oder nicht zustimmen.  DRI = digitale Rauchentwöhnungsinterventionen | | | | | |
| 11 | FC01 | Facilitating Conditions/Erleichternde Bedingungen | Ich habe die notwendige Technik (z.B. internetfähiges Smartphone oder Tablet), um DRI zu nutzen. | ordinal | Venkatesh V, Thong JY, Xu X (2012) [10], Apolinário-Hagen, Menzel, Hennemann, Salewski [7] |
|  | FC02 |  | Ich habe das notwendige technische Wissen, um DRI zu nutzen. | ordinal | Venkatesh, Thong, Xu [10], Apolinário-Hagen J, Menzel M, Hennemann S et al. (2018) [7] |
|  | FC03 |  | Wenn ich Probleme bei der Nutzung habe, weiß ich, wen ich um Hilfe bitten kann. | ordinal | Venkatesh V, Thong JY, Xu X (2012) [10], Apolinário-Hagen J, Menzel M, Hennemann S et al. (2018) Apolinário-Hagen, Menzel, Hennemann, Salewski[7] |
| Bitte geben Sie an, inwiefern Sie den folgenden Aussagen bezüglich der Befürwortung durch andere zustimmen oder nicht zustimmen. | | | | | |
| Ich denke, dass die Nutzung von digitalen Rauchentwöhnungsinterventionen von… | | | | | |
| 12 | SI01 | Social Influence/Sozialer Einfluss | …meinem Arzt/meiner Ärztin befürwortet wird. | ordinal | Zhang Y, Liu C, Luo S et al. (2019) [9] |
|  | SI02 |  | … Personen, die mir wichtig sind (z.B. Familie, Freunde), befürwortet wird. | ordinal | Zhang Y, Liu C, Luo S et al. (2019) [9] |
| Bitte geben Sie an, inwiefern Sie der folgenden Aussage bezüglich Ihrer Zahlungsbereitschaft zustimmen oder nicht zustimmen.  DRI = digitale Rauchentwöhnungsinterventionen | | | | | |
| 13 | P01 | Zahlungsbereitschaft | Für DRI wäre ich bereit, zu zahlen.  ODER  Für DRI wäre ich bei einem Rückfall bereit, zu zahlen. | ordinal | Uncovska M, Freitag B, Meister S et al. (2023) [4] |
| Bitte geben Sie an, inwiefern Sie den folgenden Aussagen bezüglich des Vertrauens in Ihre eigenen Fähigkeiten zustimmen oder nicht zustimmen.  DRI = digitale Rauchentwöhnungsinterventionen | | | | | |
| 14 | SE01 | Self Efficacy/Selbstwirksamkeit | Ich traue mir zu, DRI richtig zu nutzen, auch wenn niemand da ist, der mir zeigt, wie man sie nutzt. | ordinal | Deng Z (2013)[11] aus Uncovska M, Freitag B, Meister S et al. (2023) [4] |
|  | SE02 |  | Ich traue mir zu, Empfehlungen der DRI zur Rauchentwöhnung erfolgreich umzusetzen. | ordinal | Klaver NS, van de Klundert J, van den Broek RJGM et al. (2021) aus Uncovska M, Freitag B, Meister S et al. (2023) [4] angepasst an das Thema rauchen |
| Bitte geben Sie an, inwiefern Sie den folgenden Aussagen bezüglich Ihrer Gesundheitsbedenken zustimmen oder nicht zustimmen. | | | | | |
| 15 | PDT01 | Perceived disease threat/Wahrgenommene Gesundheitsgefährdung | Rauchen ist schädlich für die eigene Gesundheit. | ordinal | Zhang, Liu, Luo, Xie, Liu, Li, Zhou [9] aus Uncovska, Freitag, Meister, Fehring [4] |
|  | PDT02 |  | Ich bin besorgt über mein Rauchverhalten.  ODER  Während ich noch geraucht habe, war ich besorgt über mein Rauchverhalten. | ordinal | Zhang Y, Liu C, Luo S et al. (2019) [9] from Schretzlmaier P, Hecker A, Ammenwerth E (2023) [12] |
|  | PDT03 |  | Ich bin besorgt über Komplikationen, die mit dem Rauchen im Zusammenhang stehen.  ODER  Während ich noch geraucht habe, war ich besorgt über Komplikationen, die mit dem Rauchen im Zusammenhang stehen. | ordinal | Zhang Y, Liu C, Luo S et al. (2019) [9] aus Schretzlmaier P, Hecker A, Ammenwerth E (2023) [12] |
| Bitte geben Sie an, inwiefern Sie den folgenden Aussagen bezüglich Ihres Vertrauens in digitale Rauchentwöhnungsinterventionen zustimmen oder nicht zustimmen  DRI = digitale Rauchentwöhnungsinterventionen | | | | | |
| 16 | PT01 | (Perceived) Trust/Vertrauen | Ich vertraue darauf, dass DRI bewährte Methoden zur Rauchentwöhnung verwenden. | ordinal | Lee W-I, Fu H-P, Mendoza N et al. (2021) [13] from Schretzlmaier P, Hecker A, Ammenwerth E (2022) [8], eigene Übersetzung, angepasst an den Bereich Rauchen |
|  | PT03 |  | Ich vertraue darauf, dass DRI verlässliche medizinische Informationen liefern. | ordinal |  |
|  | PT03 |  | Ich vertraue darauf, dass DRI meinen Gesundheitszustand richtig interpretieren können. | ordinal |  |
| Bitte geben Sie an, inwiefern Sie den folgenden Aussagen bezüglich Ihrer Datenschutzbedenken bei der Nutzung von digitalen Rauchentwöhnungsinterventionen zustimmen oder nicht zustimmen. | | | | | |
| 17 | DP01 | Datenschutz/Data protection | Ich habe Bedenken hinsichtlich des Datenschutzes und der Datensicherheit. | ordinal | orientiert an Breinbauer M, Jansky M (2023) [14], New Item |
|  | DP02 |  | Ich habe Bedenken hinsichtlich einer Überwachung durch Dritte. | ordinal |  |
|  | DP03 |  | Ich habe Bedenken vor der Weitergabe sensibler Daten an Dritte | ordinal |  |

**Teil 4: Akzeptanz von digitalen Rauchentwöhnungsinterventionen**

Single-choice, 5-stufige Likert Skala, bestehend aus den Optionen "Stimme überhaupt nicht zu“(1), "Stimme nicht zu“(2), "Weder noch“(3), "Stimme zu“(4), "Stimme voll und ganz zu“(5), ergänzt durch die Möglichkeit "keine Antwort“(0) -> Skala ordinal

**Bitte geben Sie an, inwiefern Sie den folgenden Aussagen bezüglich Ihrer Akzeptanz zustimmen oder nicht zustimmen.
DRI = digitale Rauchentwöhnungsinterventionen**

| Nr. | Codierung | Variable | Aussage | Skala | Quelle |
| --- | --- | --- | --- | --- | --- |
| 18 | BI01 | Behavioral Intention/Akzeptanz | Ich kann mir vorstellen, DRI (wieder) zu verwenden.  ODER  Ich kann mir vorstellen, DRI bei einem Rückfall (wieder) zu verwenden. | ordinal | Venkatesh, Morris, Davis (2003) [15], Venkatesh V, Thong JY, Xu X (2012) [10] |
|  | BI02 |  | Ich beabsichtige, DRI (wieder) zu verwenden.  ODER  Ich beabsichtige, DRI bei einem Rückfall (wieder) zu verwenden. | ordinal | Venkatesh, Morris, Davis (2003) [15], Venkatesh V, Thong JY, Xu X (2012) [10] |
|  | BI03 |  | Ich beabsichtige, DRI regelmäßig (wieder) zu verwenden.  ODER  Ich beabsichtige, DRI regelmäßig bei einem Rückfall (wieder) zu verwenden. | ordinal | Venkatesh, Morris, Davis (2003) [15], Venkatesh V, Thong JY, Xu X (2012) [10] |
| 19 | DS01 | DiGA-Status | Ich wäre eher bereit, eine DRI (wieder) zu verwenden, wenn mir diese von einem Arzt oder einer Ärztin auf Rezept verschrieben wird.  ODER  Ich wäre eher bereit, DRI bei einem Rückfall (wieder) zu verwenden, wenn mir diese von einem Arzt oder einer Ärztin auf Rezept verschrieben wird. | ordinal | Uncovska M, Freitag B, Meister S et al. (2023) [4] |
|  | DS02 |  | Ich wäre eher bereit, eine DRI (wieder) zu verwenden, wenn die Wirksamkeit wissenschaftlich nachgewiesen ist.  ODER  Ich wäre eher bereit, eine DRI bei einem Rückfall (wieder) zu verwenden, wenn die Wirksamkeit wissenschaftlich nachgewiesen ist. | ordinal | Uncovska M, Freitag B, Meister S et al. (2023) |

**Teil 5: Angaben zur Person (8 von 8)**

| Nr. | Codierung | Variable | Frage | Antwort | | Skala | Quelle |
| --- | --- | --- | --- | --- | --- | --- | --- |
| 20 | age | Alter | Bitte geben Sie Ihr Alter in Jahren an.  keine Pflichtangabe | Numeric Input | | Metrisch | Uncovska M, Freitag B, Meister S et al. (2023) [4] |
| 21 | sex | Geschlecht | Bitte geben Sie Ihr Geschlecht an.  keine Pflichtangabe | Single Choice   1. weiblich 2. männlich 3. divers 4. keine Antwort | | nominal | BisQuits T0 Fragebogen, Rupp A, Blank J, Ehmann M et al. (2014) [2] |
| 22 | gkpol | Wohnort | Bitte wählen Sie aus, was auf Ihren Wohnort zutrifft.  keine Pflichtangabe | Single Choice   1. Landgemeinde (unter 5.000 Einwohner) 2. Kleinstadt (5.000 bis unter 20.000 Einwohner) 3. Mittelstadt (20.000 bis unter 100.000 Einwohner) 4. Großstadt (ab 100.000 Einwohner) 5. keine Antwort | | Metrisch | Einteilung Bundesinstitut für Bau-, Stadt- und Raumforschung [16] |
| 23 | schule | Schulabschluss | Bitte geben Sie Ihren höchsten Schulabschluss?  keine Pflichtangabe | Single Choice   1. kein allgemeinbildender Schulabschluss 2. Haupt-/Volksschulabschluss 3. Realschulabschluss/Mittlere Reife/Polytechn. Oberschule 4. Fachhochschulreife 5. Abitur/Allgemeine Hochschulreife 6. anderer Schulabschluss (z. B. Ausland) 7. keine Antwort | | ordinal | Aus Baseline-Fragebogen der DEBRA Studie [1]  Erhebung Marktforschungsinstitut |
| 24 | berab | Berufsabschluss | Bitte geben Sie Ihren höchsten Berufsabschluss?  keine Pflichtangabe | Single Choice   1. kein Berufsabschluss 2. abgeschlossene Lehre/beruflich-betriebliche Ausbildung 3. Ausbildung an Fach-, Meister-, Technikerschule, Berufs-/Fachakademie 4. Fachhochschulabschluss 5. Universitätsabschluss 6. anderer beruflicher Abschluss 7. keine Antwort | ordinal | | Aus Baseline-Fragebogen der DEBRA Studie [1]  Erhebung Marktforschungsinstitut |
| 25 | berst | Berufliche Stellung | Bitte geben Sie Ihre aktuelle beruflichen Stellung an.  keine Pflichtangabe | Single Choice   1. Schüler/in 2. Auszubildene/r 3. Student/in 4. Arbeiter/in (überwiegend körperliche Arbeit) 5. Angestellter® (überwiegend geistige Arbeit) 6. Beamte® 7. Selbstständige® 8. Arbeitslose/r 9. Mutterschafts-/Erziehungsurlaub, Elternzeit, sonst. Beurlaubung 10. Hausfrau/Hausmann 11. Rentner/in 12. andere berufliche Stellung 13. keine Antwort | ordinal | | Aus Baseline-Fragebogen der DEBRA Studie [1]  Erhebung Marktforschungsinstitut  (zusammengefasst) |
| 26 | afam | Familienstand | Bitte geben Sie Ihren derzeitigen Familienstand an.  keine Pflichtangabe | Single Choice   1. ledig 2. verheiratet/eingetragene Lebenspartnerschaft 3. verwitwet 4. geschieden 5. keine Angabe | | nominal | Aus Baseline-Fragebogen der DEBRA Studie [1]  Erhebung Marktforschungsinstitut |

**Vielen Dank für Ihre Teilnahme an der Befragung!**
Ihre Antworten helfen, das Nutzungsverhalten und die Erfahrungen der deutschen Bevölkerung mit digitalen Rauchentwöhnungsinterventionen zu untersuchen und Faktoren zu identifizieren, die die Nutzerakzeptanz beeinflussen. Auf diese Weise können Nutzungsbarrieren erkannt und Maßnahmen zur Erhöhung der Nutzerakzeptanz abgeleitet werden.

Für weitere Fragen oder um die Ergebnisse der Befragung zu erhalten, wenden Sie sich bitte an folgende E-Mail-Adresse: [mail.com](mailto:franziska.theile@wig2.de)

[Hier gelangen Sie zur Teilnahme am Gewinnspiel!](https://bildungsportal.sachsen.de/umfragen/limesurvey/index.php/473551?lang=de)

References

1. Kotz D, Kastaun S, Klosterhalfen S (2019) OSF | DEBRA-II Baseline Fragebogen_v50_KH.pdf. https://osf.io/tnj83. Accessed 19 Jul 2024

2. Rupp A, Blank J, Ehmann M et al. (2014) Basisdaten der „Brief Intervention Study for Quitting Smoking“ – BisQuits. Pneumologie 68. https://doi.org/10.1055/s-0034-1367889

3. Latza U, Hoffmann W, Terschüren C et al. Erhebung, Quantifizierung und Analyse der Rauchexposition in epidemiologischen Studien. Robert Koch-Inst, Berlin

4. Uncovska M, Freitag B, Meister S et al. (2023) Patient Acceptance of Prescribed and Fully Reimbursed mHealth Apps in Germany: An UTAUT2-based Online Survey Study. J Med Syst 47:14. https://doi.org/10.1007/s10916-023-01910-x

5. Breil B, Kremer L, Hennemann S et al. (2019) Acceptance of mHealth Apps for Self-Management Among People with Hypertension. Stud Health Technol Inform 267:282–288. https://doi.org/10.3233/SHTI190839

6. Hennemann S, Beutel ME, Zwerenz R (2016) Drivers and Barriers to Acceptance of Web-Based Aftercare of Patients in Inpatient Routine Care: A Cross-Sectional Survey. Journal of Medical Internet Research 18:e337. https://doi.org/10.2196/jmir.6003

7. Apolinário-Hagen J, Menzel M, Hennemann S et al. (2018) Acceptance of Mobile Health Apps for Disease Management Among People With Multiple Sclerosis: Web-Based Survey Study. JMIR Form Res 2:e11977. https://doi.org/10.2196/11977

8. Schretzlmaier P, Hecker A, Ammenwerth E (2022) Extension of the Unified Theory of Acceptance and Use of Technology 2 model for predicting mHealth acceptance using diabetes as an example: a cross-sectional validation study. BMJ Health Care Inform 29:e100640. https://doi.org/10.1136/bmjhci-2022-100640

9. Zhang Y, Liu C, Luo S et al. (2019) Factors Influencing Patients' Intentions to Use Diabetes Management Apps Based on an Extended Unified Theory of Acceptance and Use of Technology Model: Web-Based Survey. Journal of Medical Internet Research 21:e15023. https://doi.org/10.2196/15023

10. Venkatesh V, Thong JY, Xu X (2012) Consumer Acceptance and Use of Information technology: extending The Unfified theory of Acceptance and Use of technology

11. Deng Z (2013) Understanding public users' adoption of mobile health service. International Journal of Mobile Communications 11:351. https://doi.org/10.1504/IJMC.2013.055748

12. Schretzlmaier P, Hecker A, Ammenwerth E (2023) Predicting mHealth Acceptance Using the UTAUT2 Technology Acceptance Model: A Mixed-Methods Approach. Stud Health Technol Inform 301:26–32. https://doi.org/10.3233/SHTI230007

13. Lee W-I, Fu H-P, Mendoza N et al. (2021) Determinants Impacting User Behavior towards Emergency Use Intentions of m-Health Services in Taiwan. Healthcare 9:535. https://doi.org/10.3390/healthcare9050535

14. Breinbauer M, Jansky M (2023) Gesundheits-Apps in der hausärztlichen Versorgung. Präv Gesundheitsf. https://doi.org/10.1007/s11553-023-01057-0

15. Venkatesh V, Morris MG, Davis FD (2003) User Acceptance of Information Technology: Toward a Unified View. MIS Quarterly 27:425. https://doi.org/10.2307/30036540

16. Bundesinstitut für Bau-, Stadt- und Raumforschung im Bundesamt für Bauwesen und Raumordnung (2023) Raumbeobachtung - Stadt- und Gemeindetypen in Deutschland. https://www.bbsr.bund.de/BBSR/DE/forschung/raumbeobachtung/Raumabgrenzungen/deutschland/gemeinden/StadtGemeindetyp/StadtGemeindetyp.html. Accessed 21 Feb 2024
