# Supplementary material for: Understanding acceptance of digital smoking cessation interventions: user behavior, key influencing factors, and the role of reimbursement
Source: BMC Public Health. 2025 Dec 12;26:218. doi: 10.1186/s12889-025-25472-4 (PMC12817400; doi:10.1186/s12889-025-25472-4)
Supplement: Supplementary file 2 — supplementary material 2. [file 12889_2025_25472_MOESM2_ESM.docx]

(2) Questionnaire - English version (translated)

! ***Note:*** This questionnaire represents a translated and contextually adapted version of the original German instrument. For items without existing translations, a forward translation was performed in accordance with established methodological principles, and the wording was reviewed and refined within the research team to ensure linguistic clarity and conceptual consistency. The English version has not undergone formal linguistic validation or back-translation and is provided solely for documentation and illustrative purposes. It is not intended for use in further studies.

**Part 1: Information on smoking behaviour (1 of 8)**

**Please note that the following questions refer to tobacco cigarettes and not to electronic cigarettes.**

| No. | Coding | Variable | Question | Answer options | Scale level | References |
| --- | --- | --- | --- | --- | --- | --- |
| 01 | sok | Smoking | Which of the following statements applies to you the most? | Single Choice   1. I smoke every day. 2. I do not smoke every day, but at least 1 packet of cigarettes per month. 3. I only smoke on certain occasions (e.g. social events). 4. I have given up smoking completely in the last year. 5. I quit smoking completely more than a year ago. 6. I have never smoked. -> Survey completed, use of quotas | nominal | From the baseline questionnaire of the DEBRA study [1]  Survey framework:   - Conventional tobacco cigarettes - smokers (1) and (2) - occasional smokers (3) - Ex-Smokers (4) and (5) - Non-Smokers (6) |
| 02 | tr01 | Start smoking | In which year did you start smoking for the first time?  1920<tr01<2024  Not mandatory | Numeric Input | metric | From the baseline questionnaire of the DEBRA study [1]  BisQuits study asks about age at start of smoking [2] |
|  | tr02 | Smokers | [If answered 01 with (5)] In which year did you stop smoking completely? smokers  tr02<tr02<2023  not mandatory | Numeric Input | metric | From questions Active smoking - short version (adults) by smokers [3] |
| 03 | aw | Wish to quit smoking | [if answered 01 with (1) OR (2) OR (3)] How strong is your current desire to stop smoking? | Not at all strong (0)  Extremely strong (10)  Visual analogue scale from 0 to 10 | ordinal | From BisQuit's T0 questionnaire of Rupp, Blank, Ehmann, Pousset, Mühlig, Sehl, Fuchs, Rüther, Linhardt, Grah, Kreuter Rupp A, Blank J, Ehmann M et al. (2014) [2] |
| 04 | av01 | Attempts to stop smoking | [if answered 01 with (1) OR (2) OR (3)] How many **serious** attempts have you made thus far to stop smoking in total?  av01>0 | Numeric Input | metric | From BisQuit's T0 questionnaire of Rupp A, Blank J, Ehmann M et al. (2014) [2] |
|  | av02 |  | [if answered 01 with (4) OR (5)] How many serious attempts have you made in total to stop smoking?  avo2>1 | Numeric Input | metric | From BisQuit's T0 questionnaire of Rupp A, Blank J, Ehmann M et al. (2014)[2] |

**Part 2: Experiences with digital health interventions for smoking cessation (2 of 8)**

Digital smoking cessation interventions (DSCIs) are mobile apps designed to help smokers overcome their tobacco addiction and quit smoking permanently (e.g., SmokeFree - Quit Smoking, QuitNow!). Typical functions include a stop-smoking programme, motivational aids, distraction options for smoking cravings and information on health status.

| No. | Coding | Variable | Question | Answer options | Scale | References |
| --- | --- | --- | --- | --- | --- | --- |
| 05 | exp | experience | Have you ever used a digital smoking cessation intervention? | Single Choice   1. Yes 2. No | nominal | New Item |
| 06 | app | Digital health interventions | [If answers 05 with (1)]: Which digital smoking cessation intervention(s) have you used? | Multiple Choice   1. NichtraucherHelden-App 2. Smoke Free 3. Rauchen aufhören – Kwit (iOs)/Kwit – Rauchen war gestern (Android) 4. QuitNow! 5. Rauchen aufhören – Easy Quit 6. Rauchfrei Lite (iOs)/Rauchfrei – aufhören zu rauchen (Android) 7. Rauchen aufhören mit Flamy 8. QuitSure: Rauchen aufhören 9. TK-RauchFrei 10. Other | nominal | Selection is based on the download figures and the number of reviews in the Google Play Store and Apple App Store  ATTENTION: names differ in the stores |
| 07 | tn | Period of use | [If answered 05 with (1)]: How long have you used the digital smoking cessation intervention?  Note: If you have used several interventions, please answer the question regarding the application that you have used the longest. | Single Choice   1. 1 to 3 days 2. over 3 days up to 1 week 3. over 1 week up to 1 month 4. over 1 month up to 3 months 5. over 3 months up to 6 months 6. over 6 months up to 12 months 7. longer than 12 months | metric | Question comes from the study by Uncovska M, Freitag B, Meister S et al. (2023) [4][5]  Answer categories are based on the duration of use of Smoke Free and the NichtraucherHelden-App |
| 08 | rf01 | Smokefree | [If answered 01 with (1) OR (2) OR (3) AND 05 with (1)] How many months were you smoke-free after you stopped using the digital smoking cessation intervention?  Note:   - If you have been smoke-free for less than 1 month, please enter 0. - If you used several interventions, please answer the question regarding the application you have used the longest.   Not mandatory | Numeric Input, integer, 2 digits  _ months | metric | Based on the BISQUITS T0 questionnaire from Rupp A, Blank J, Ehmann M et al. (2014) [2] |
|  | rf02 | Rauchfrei | [If answered 1 with (4) OR (5) AND 05 with (1)] Have you quit smoking sustainably with the help of a digital smoking cessation intervention? | Single Choice   1. Yes 2. No | nominal | NEW ITEM |

Single-choice, 5-point Likert scale, consisting of the options ‘strongly disagree’(1), ‘disagree’(2), ‘neither’(3), “agree”(4), and ‘strongly agree’(5), supplemented by the option ‘no answer’(0) 🡪 ordinal scale

Please note that the wording of the following statements was deliberately designed so that they can be answered equally by smokers and former smokers, with or without previous experience of use.

| No. | Coding | Variable | Item | Scale | Reference |
| --- | --- | --- | --- | --- | --- |
| Please indicate the extent to which you agree or disagree with the following statements regarding your user experience with digital smoking cessation interventions.  Below you will find a series of statements. If you fully agree with a statement, please select the penultimate option. If you do not agree with the statement at all, please select the first option. You can use the options in-between to grade your assessment. Your judgement is of crucial importance for the research work. However, if you are unable or unwilling to assess a statement, please select the last option.  DSCI = Digital Smoking Cessation Intervention | | | | | |
| 09 | UEX01 | User experience | DSCI helps you to stop smoking. | ordinal | Uncovska M, Freitag B, Meister S et al. (2023) [4] |
|  | UEX02 |  | DSCI helps to deal with smoke odours. | ordinal | Uncovska M, Freitag B, Meister S et al. (2023) [4]  Breil B, Kremer L, Hennemann S et al. (2019) [6], Hennemann S, Beutel ME, Zwerenz R (2016) [7] |
|  | UEX03 |  | DSCI improve your own health and well-being. | ordinal | Apolinário-Hagen J, Menzel M, Hennemann S et al. (2018) [8] |
|  | UEX04 |  | DSCIs are easy to use and understand. | ordinal | Schretzlmaier P, Hecker A, Ammenwerth E (2022) [9], Hennemann S, beutel ME, Zwerenz R (2016) [7], Zhang Y, Liu C, Luo S et al. (2019) [10] |
|  | UEX04 |  | DSCIs are easy to learn how to use. | ordinal | Schretzmaier P, Hecker A, Ammenwerth E (2022) [9], Hennemann S, Beutel ME, Zwerenz R (2016) [7], Zhang Y, Liu C, Luo S et al. (2019) [10] |

**Part 3: Factors influencing acceptance (3,4,5,6 of 8)**

Single-choice, 5-point Likert scale, consisting of the options ‘Strongly disagree’(1), ‘Disagree’(2), ‘Neither’(3), “Agree”(4), and ‘Strongly agree’(5), supplemented by the option ‘No answer’(0) -> ordinal scale

| No. | Coding | Variable | Item | Scale | Reference |
| --- | --- | --- | --- | --- | --- |
| Please indicate the extent to which you agree or disagree with the following statements regarding your user expectations.  Below you will find a series of statements. If you fully agree with a statement, please select the second last option. If you do not agree with the statement at all, please select the first option. You can use the options in-between to grade your assessment. Your judgement is of crucial importance for the research work. However, if you are unable or unwilling to assess a statement, please select the last option. | | | | | |
| I expect that digital smoking cessation interventions... | | | | | |
| 09 | PE01 | Performance Expectancy | ... help people to stop smoking. | ordinal | Uncovska M, Freitag B, Meister S et al. (2023)[4] |
|  | PE02 |  | … help deal with smoking urges. | ordinal | Uncovska M, Freitag B, Meister S et al. (2023) [4]  Breil B, Kremer L, Hennemann S et al. (2019) [6], Hennemann S, Beutel ME, Zwerenz R (2016) [7] |
|  | PE03 |  | ...will improve your own health and well-being. | ordinal | Apolinário-Hagen J, Menzel M, Hennemann S et al. (2018) [8] |
| 10 | EE01 | Effort Expectancy | ...to be easy to use and understand. | ordinal | Schretzlmaier P, Hecker A, Ammenwerth E (2022) [9], Hennemann S, Beutel ME, Zwerenz R (2016) [7], Zhang Y, Liu C, Luo S et al. (2019) [10] |
|  | EE02 |  | ...easy to learn how to use. | ordinal | Schretzmaier P, Hecker A, Ammenwerth E (2022) [9], Hennemann S, Beutel ME, Zwerenz R (2016) [7], Zhang Y, Liu C, Luo S et al. (2019) [10] |
| Please indicate the extent to which you agree or disagree with the following statements regarding your utilization requirements.  DSCI = Digital Smoking Cessation Intervention | | | | | |
| 11 | FC01 | Facilitating conditions | I have the necessary technology (e.g. internet-enabled smartphone or Tablet) to use DSCI. | ordinal | Venkatesh V, Thong JY, Xu X (2012) [11], Apolinário-Hagen, Menzel, Hennemann, Salewski [8] |
|  | FC02 |  | I have the technical knowledge necessary to use DSCI. | ordinal | Venkatesh, Thong, Xu [11], Apolinário-Hagen J, Menzel M, Hennemann S et al. (2018) [8] |
|  | FC03 |  | If I have problems using DSCI, I know who I can ask for help. | ordinal | Venkatesh V, Thong JY, Xu X (2012) [11], Apolinário-Hagen J, Menzel M, Hennemann S et al. (2018) [8] |
| Please indicate the extent to which you agree or disagree with the following statements regarding endorsement by others. | | | | | |
| I think that the use of digital smoking cessation interventions... | | | | | |
| 12 | SI01 | Social Influence | … is supported by my doctor. | ordinal | Zhang Y, Liu C, Luo S et al. (2019) [10] |
|  | SI02 |  | … is supported by people who are important to me (e.g. family, friends). | ordinal | Zhang Y, Liu C, Luo S et al. (2019) [10] |
| Please indicate to what extent you agree or disagree with the following statement regarding your willingness to pay.  DSCI = Digital Smoking Cessation Intervention | | | | | |
| 13 | WP01 | Willingness to pay | I would be willing to pay for DSCI.  OR  I would be willing to pay for DRI in the event of a relapse. | ordinal | Uncovska M, Freitag B, Meister S et al. (2023) [4] |
| Please indicate the extent to which you agree or disagree with the following statements regarding confidence in your own abilities.  DSCI = Digital Smoking Cessation Intervention | | | | | |
| 14 | SE01 | Self-Efficacy | I have the confidence to use DSCI properly, even if there is no one around to show me how to use it. | ordinal | Deng Z (2013) [12] from Uncovska M, Freitag B, Meister S et al. (2023) [4] |
|  | SE02 |  | I am confident that I can successfully implement DSCIs recommendations for smoking cessation. | ordinal | Klaver NS, van de Klundert J, van den Broek RJGM et al. (2021) [13] from Uncovska M, Freitag B, Meister S et al. (2023) [4] adapted to the topic of smoking |
| Please indicate the extent to which you agree or disagree with the following statements regarding your health concerns. | | | | | |
| 15 | PDT01 | Perceived disease threat | Smoking is harmful to your health. | ordinal | Zhang, Liu, Luo, Xie, Liu, Li, Zhou [10] aus Uncovska, Freitag, Meister, Fehring [4] |
|  | PDT02 |  | I am concerned about my smoking behavior.  OR  While I was still smoking, I was concerned about my smoking behavior. | ordinal | Zhang Y, Liu C, Luo S et al. (2019) [10] from Schretzlmaier P, Hecker A, Ammenwerth E [14] |
|  | PDT03 |  | I am concerned about complications associated with smoking.  OR  While I was still smoking, I was concerned about complications associated with smoking. | ordinal | Zhang Y, Liu C, Luo S et al. (2019) [10] from Schretzlmaier P, Hecker A, Ammenwerth E (2023) Schretzlmaier, Hecker, Ammenwerth[14] |
| Please indicate the extent to which you agree or disagree with the following statements regarding your confidence in digital smoking cessation interventions.  DSCI = Digital Smoking Cessation Intervention | | | | | |
| 16 | PT01 | (Perceived) Trust | I trust that DSCI uses proven methods for smoking cessation. | ordinal | Lee W-I, Fu H-P, Mendoza N et al. (2021) [15] from Schretzlmaier P, Hecker A, Ammenwerth E (2022) [9], own translation, adapted to the area of smoking |
|  | PT03 |  | I trust DSCI to provide reliable medical information. | ordinal |  |
|  | PT03 |  | I trust that DSCI can interpret my state of health correctly. | ordinal |  |
| Please indicate the extent to which you agree or disagree with the following statements regarding your privacy concerns when using digital smoking cessation interventions. | | | | | |
| 17 | DP01 | Data protection | I have concerns about data protection and data security. | ordinal | Orientated to Breinbauer M, Jansky M (2023) [16], New Item |
|  | DP02 |  | I have concerns about monitoring by third parties. | ordinal |  |
|  | DP03 |  | I have concerns about the disclosure of sensitive data to third parties | ordinal |  |

**Part 4: Acceptance of digital smoking cessation interventions**

Single-choice, 5-point Likert scale, consisting of the options ‘Strongly disagree’(1), ‘Disagree’(2), ‘Neither’(3), “Agree”(4), and ‘Strongly agree’(5), supplemented by the option ‘No answer’(0) -> ordinal scale

**Please indicate the extent to which you agree or disagree with the following statements regarding your acceptance.
DSCI = Digital Smoking Cessation Intervention**

| No. | Coding | Variable | Item | Scale | Reference |
| --- | --- | --- | --- | --- | --- |
| 18 | BI01 | Behavioral Intention | I intend to use DSCI (again).  OR  I intend to use DSCI (again) in the event of a relapse. | ordinal | Venkatesh, Morris, Davis (2003) [17], Venkatesh V, Thong JY, Xu X (2012) [11] |
|  | BI02 |  | I predict I would use DSCI (again).  OR  I predict I would use DSCI (again) in the event of a relapse. | ordinal | Venkatesh, Morris, Davis (2003) [17], Venkatesh V, Thong JY, Xu X (2012) [11] |
|  | BI03 |  | I plan to use DSCI (again) in the event of a relapse.  OR  I plan to use DSCI regularly in the event of a relapse (again). | ordinal | Venkatesh, Morris, Davis (2003) [17], Venkatesh V, Thong JY, Xu X (2012) [11] |
| 19 | DS01 | DiGA Status | I would be more willing to use a DSCI (again) if it was prescribed to me by a doctor.  OR  I would be more willing to use DSCI (again) in the event of a relapse if I was prescribed it by a doctor. | ordinal | Uncovska M, Freitag B, Meister S et al. (2023) [4] |
|  | DS02 |  | I would be more willing to use a DSCI (again) if the effectiveness is scientifically proven.  OR  I would be more willing to use a DSCI in a relapse (again) if the efficacy is scientifically proven. | ordinal | Uncovska M, Freitag B, Meister S et al. (2023) [4] |

**Part 5: Personal data (8 of 8)**

| No. | Coding | Variable | Question | Answer Options | Scale | Reference |
| --- | --- | --- | --- | --- | --- | --- |
| 20 | age | age | Please enter your age in years.  not mandatory | Numeric Input | metric | Uncovska M, Freitag B, Meister S et al. (2023) [4] |
| 21 | sex | sex | Please enter your gender.  not mandatory | Single Choice   1. female 2. male 3. diverse 4. no answer | nominal | BisQuits T0 questionnaire, Rupp A, Blank J, Ehmann M et al. (2014) [2] |
| 22 | gkpol | Place of residence | Please select what applies to your place of residence.  not mandatory | Single Choice   1. Rural municipality (under 5,000 inhabitants) 2. Small town (5,000 to under 20,000 inhabitants) 3. Medium-sized city (20,000 to under 100,000 inhabitants) 4. Large city (100,000 inhabitants or more) 5. No answer | metric | Classification of Bundesinstitut für Bau-, Stadt- und Raumforschung [18] |
| 23 | schule | School leaving qualification | Please enter your highest school-leaving qualification?  Not mandatory | Single Choice   1. no general school leaving certificate 2. secondary school/elementary school 3. Middle school/intermediate school/polytechnic secondary school 4. University of applied sciences 5. Abitur/general higher education entrance qualification 6. Other school leaving certificate (e.g. abroad) 7. No answer | ordinal | From the baseline questionnaire of the DEBRA study [1]  Market research institute survey |
| 24 | berab | Professional qualification | Please enter your highest professional qualification?  Not mandatory | Single Choice   1. No professional qualification 2. Completed apprenticeship/vocational/company training 3. Training at vocational, master craftsman, technical school, vocational/technical academy 4. University of Applied Sciences degree 5. University degree 6. Other professional qualification 7. No answer | ordinal | From the baseline questionnaire of the DEBRA study [1]  Market research institute survey |
| 25 | berst | Professional position | Please indicate your current professional position.  not mandatory | Single Choice   1. Pupil 2. Apprentice 3. Student 4. Worker (mainly physical labor) 5. Employee (mainly mental labor) 6. Civil servant 7. Self-employed 8. Unemployed 9. Maternity/parental leave, parental leave, other leave of absence 10. Housewife/househusband 11. Pensioner 12. Other professional position 13. No answer | ordinal | From the baseline questionnaire of the DEBRA study [1]  Survey by market research institute  (summarized) |
| 26 | afam | family status | Please enter your current marital status.  not mandatory | Single Choice   1. single 2. married/registered civil partnership 3. widowed 4. divorced 5. not answer | nominal | From the baseline questionnaire of the DEBRA study [1]  Market research institute survey |

**Thank you for taking part in the survey!**

**Your answers will help us investigate the usage behaviour and experiences of the German population with digital smoking cessation interventions and identify factors that influence user acceptance. In this way, barriers to use can be recognized, and measures to increase user acceptance can be derived.**

For further questions or to receive the results of the survey, please contact the following e-mail address: mail.com

Click here to enter the competition!

References

1. Kotz D, Kastaun S, Klosterhalfen S (2019) OSF | DEBRA-II Baseline Fragebogen_v50_KH.pdf. https://osf.io/tnj83. Accessed 19 Jul 2024

2. Rupp A, Blank J, Ehmann M et al. (2014) Basisdaten der „Brief Intervention Study for Quitting Smoking“ – BisQuits. Pneumologie 68. https://doi.org/10.1055/s-0034-1367889

3. Latza U, Hoffmann W, Terschüren C et al. Erhebung, Quantifizierung und Analyse der Rauchexposition in epidemiologischen Studien. Robert Koch-Inst, Berlin

4. Uncovska M, Freitag B, Meister S et al. (2023) Patient Acceptance of Prescribed and Fully Reimbursed mHealth Apps in Germany: An UTAUT2-based Online Survey Study. J Med Syst 47:14. https://doi.org/10.1007/s10916-023-01910-x

5. Uncovska M, Freitag B, Meister S et al. (2023) Appendix 2: Review of existing literature on mHealth acceptance

6. Breil B, Kremer L, Hennemann S et al. (2019) Acceptance of mHealth Apps for Self-Management Among People with Hypertension. Stud Health Technol Inform 267:282–288. https://doi.org/10.3233/SHTI190839

7. Hennemann S, Beutel ME, Zwerenz R (2016) Drivers and Barriers to Acceptance of Web-Based Aftercare of Patients in Inpatient Routine Care: A Cross-Sectional Survey. Journal of Medical Internet Research 18:e337. https://doi.org/10.2196/jmir.6003

8. Apolinário-Hagen J, Menzel M, Hennemann S et al. (2018) Acceptance of Mobile Health Apps for Disease Management Among People With Multiple Sclerosis: Web-Based Survey Study. JMIR Form Res 2:e11977. https://doi.org/10.2196/11977

9. Schretzlmaier P, Hecker A, Ammenwerth E (2022) Extension of the Unified Theory of Acceptance and Use of Technology 2 model for predicting mHealth acceptance using diabetes as an example: a cross-sectional validation study. BMJ Health Care Inform 29:e100640. https://doi.org/10.1136/bmjhci-2022-100640

10. Zhang Y, Liu C, Luo S et al. (2019) Factors Influencing Patients' Intentions to Use Diabetes Management Apps Based on an Extended Unified Theory of Acceptance and Use of Technology Model: Web-Based Survey. Journal of Medical Internet Research 21:e15023. https://doi.org/10.2196/15023

11. Venkatesh V, Thong JY, Xu X (2012) Consumer Acceptance and Use of Information technology: extending The Unfified theory of Acceptance and Use of technology

12. Deng Z (2013) Understanding public users' adoption of mobile health service. International Journal of Mobile Communications 11:351. https://doi.org/10.1504/IJMC.2013.055748

13. Klaver NS, van de Klundert J, van den Broek RJGM et al. (2021) Relationship Between Perceived Risks of Using mHealth Applications and the Intention to Use Them Among Older Adults in the Netherlands: Cross-sectional Study. JMIR mHealth and uHealth 9:e26845. https://doi.org/10.2196/26845

14. Schretzlmaier P, Hecker A, Ammenwerth E (2023) Predicting mHealth Acceptance Using the UTAUT2 Technology Acceptance Model: A Mixed-Methods Approach. Stud Health Technol Inform 301:26–32. https://doi.org/10.3233/SHTI230007

15. Lee W-I, Fu H-P, Mendoza N et al. (2021) Determinants Impacting User Behavior towards Emergency Use Intentions of m-Health Services in Taiwan. Healthcare 9:535. https://doi.org/10.3390/healthcare9050535

16. Breinbauer M, Jansky M (2023) Gesundheits-Apps in der hausärztlichen Versorgung. Präv Gesundheitsf. https://doi.org/10.1007/s11553-023-01057-0

17. Venkatesh V, Morris MG, Davis FD (2003) User Acceptance of Information Technology: Toward a Unified View. MIS Quarterly 27:425. https://doi.org/10.2307/30036540

18. Bundesinstitut für Bau-, Stadt- und Raumforschung im Bundesamt für Bauwesen und Raumordnung (2023) Raumbeobachtung - Stadt- und Gemeindetypen in Deutschland. https://www.bbsr.bund.de/BBSR/DE/forschung/raumbeobachtung/Raumabgrenzungen/deutschland/gemeinden/StadtGemeindetyp/StadtGemeindetyp.html. Accessed 21 Feb 2024
