# Supplementary material for: Understanding acceptance of digital smoking cessation interventions: user behavior, key influencing factors, and the role of reimbursement
Source: BMC Public Health. 2025 Dec 12;26:218. doi: 10.1186/s12889-025-25472-4 (PMC12817400; doi:10.1186/s12889-025-25472-4)
Supplement: Supplementary file 3 — supplementary material 3. [file 12889_2025_25472_MOESM3_ESM.docx]

(3) Factors influencing acceptance


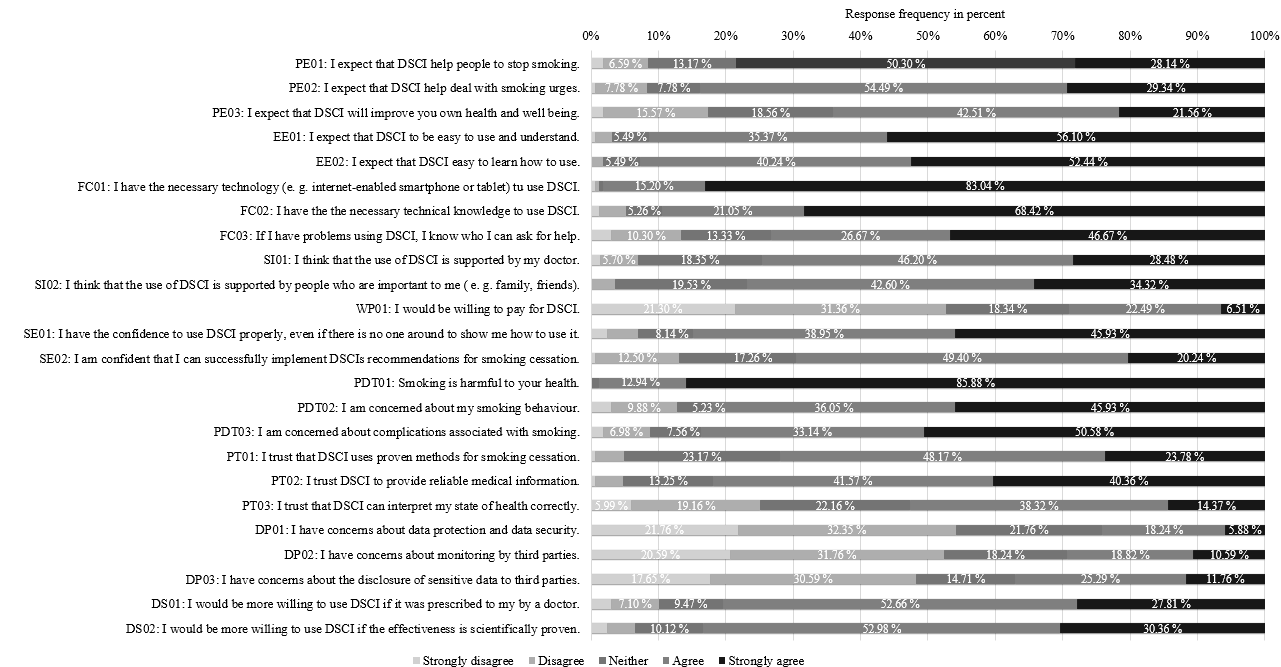


Figure 3 Distribution of Responses on Acceptance Factors for DSCI in Percent (response category 'no answer' was excluded from analysis)
